# A Letter

**Published:** 1865-02

**Authors:** 


### A LETTER.

——— City, Ill., 1865, Feb. 17.

Mr. ———

received your Bill A gance me hope it is all rite, as I tole you to send your fool A Count A gance me—I hant had time to look at it—4 here now from Indana for hole sets And half sets—used 3 sets of your teeth A redy I fere they wont last long—I wood A sent you A draft yester day but I Cudent git time to go to the Bank.

in Closed you will find A draft for one hundred dollars A Corden to your Bill.

your,

I must go to work. Wm. ———

---

We give the above, a model in its way, exactly according to copy minus the name. It is amusing and vexing—laughable and mournful—ridiculous and yet solemn,—yes solemn-colly! If we have the blues and want to be cured we read this letter; if we are too jubilant and want to be brought down a few notches, we read this letter. But, seriously, it is a burning withering shame, that men of such attainments should be encouraged or even permitted to enter the profession. That man could not take a higher position in our common schools than the second reader.

We have often urged upon our professional brethren the importance and necessity of looking scrupulously to the elementary attainments of those whom they aid and encourage to enter the profession. There are many now in the profession who are very deficient in this respect, this can only be changed by an effort for their improvemnt; but for the future more care should be exercised than has been in the past.

